# Supplementary material for: The interactome of intact mitochondria by cross-linking mass spectrometry provides evidence for coexisting respiratory supercomplexes
Source: Mol Cell Proteomics. 2017 Dec 8;17(2):216–32. doi: 10.1074/mcp.RA117.000470 (PMC5795388; doi:10.1074/mcp.RA117.000470)
Supplement: Supplemental Data [file supp_RA117.000470_133922_0_supp_23978_fzffwf.zip › spectra_annotation/mito_DR_spectra_annotation/73-1-1-1-5-1.pdf]

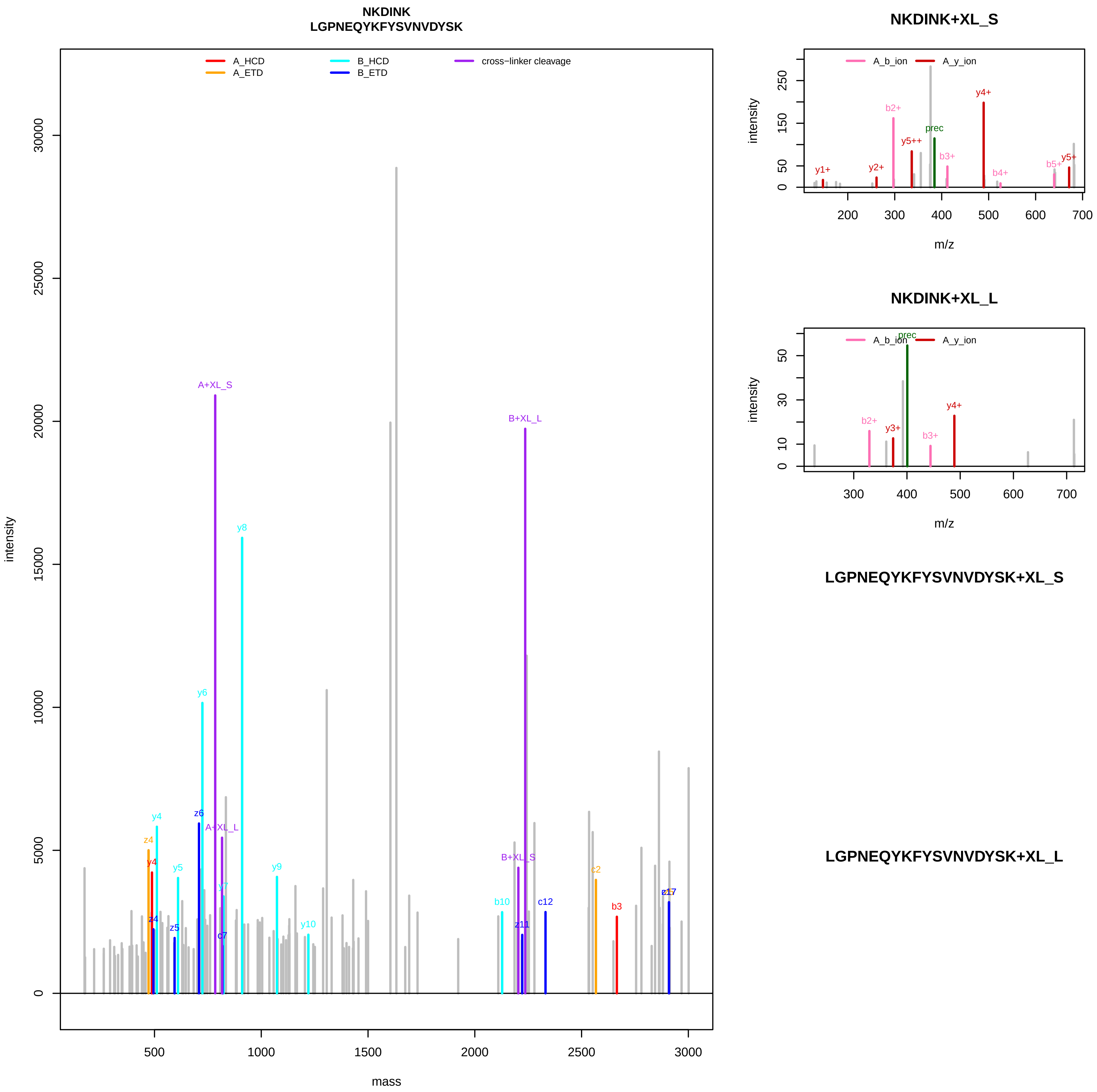
NKDINK
LGPNEQYKFYSVNVDYSK
A_HCD
A_ETD
B_HCD
B_ETD
cross−linker cleavage
intensity
mass
A+XL_S
B+XL_L
y8
y6
y4
z4
y4
y5
z6
A+XL_L
y7
y9
y10
z4
z5
c7
B+XL_S
b10
z11
c12
c2
b3
c17
z17
0
5000
10000
15000
20000
25000
30000
500
1000
1500
2000
2500
3000
NKDINK+XL_S
A_b_ion
A_y_ion
y1+
y2+
b2+
y5++
prec
b3+
y4+
b4+
b5+
y5+
m/z
NKDINK+XL_L
b2+
y3+
b3+
y4+
LGPNEQYKFYSVNVDYSK+XL_S
LGPNEQYKFYSVNVDYSK+XL_L
